# Supplementary material for: Kinkless Electronic Junction along 1D Electronic Channel Embedded in a Van Der Waals Layer
Source: Adv Sci (Weinh). 2023 Dec 7;11(3):2307831. doi: 10.1002/advs.202307831 (PMC10797480; doi:10.1002/advs.202307831)
Supplement: Supplementary file 1 — Supporting Information [file ADVS-11-2307831-s001.pdf]

ADVANCED SCIENCE
Open Access

Supporting Information

for *Adv. Sci.*, DOI 10.1002/advs.202307831

Kinkless Electronic Junction along 1D Electronic Channel Embedded in a Van Der Waals Layer

*Qirong Yao, Jae Whan Park, Choongjae Won, Sang-Wook Cheong and Han Woong Yeom**

Supporting Information

Kinkless electronic junction along 1D electronic channel embedded in a van der Waals layer

*Qirong Yao, Jae Whan Park, Choongjae Won, Sang-Wook Cheong, and Han Woong Yeom**

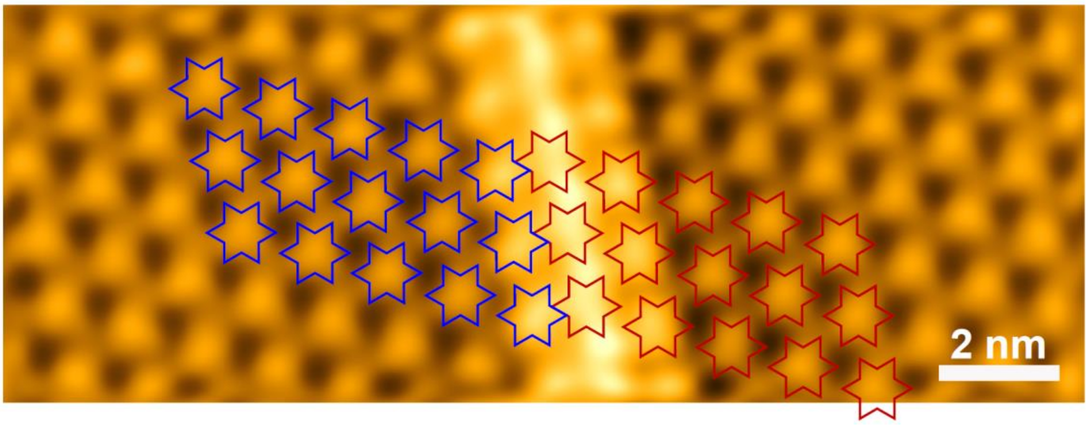

Figure S1. STM topography image with a second type of domain wall (DW-2).

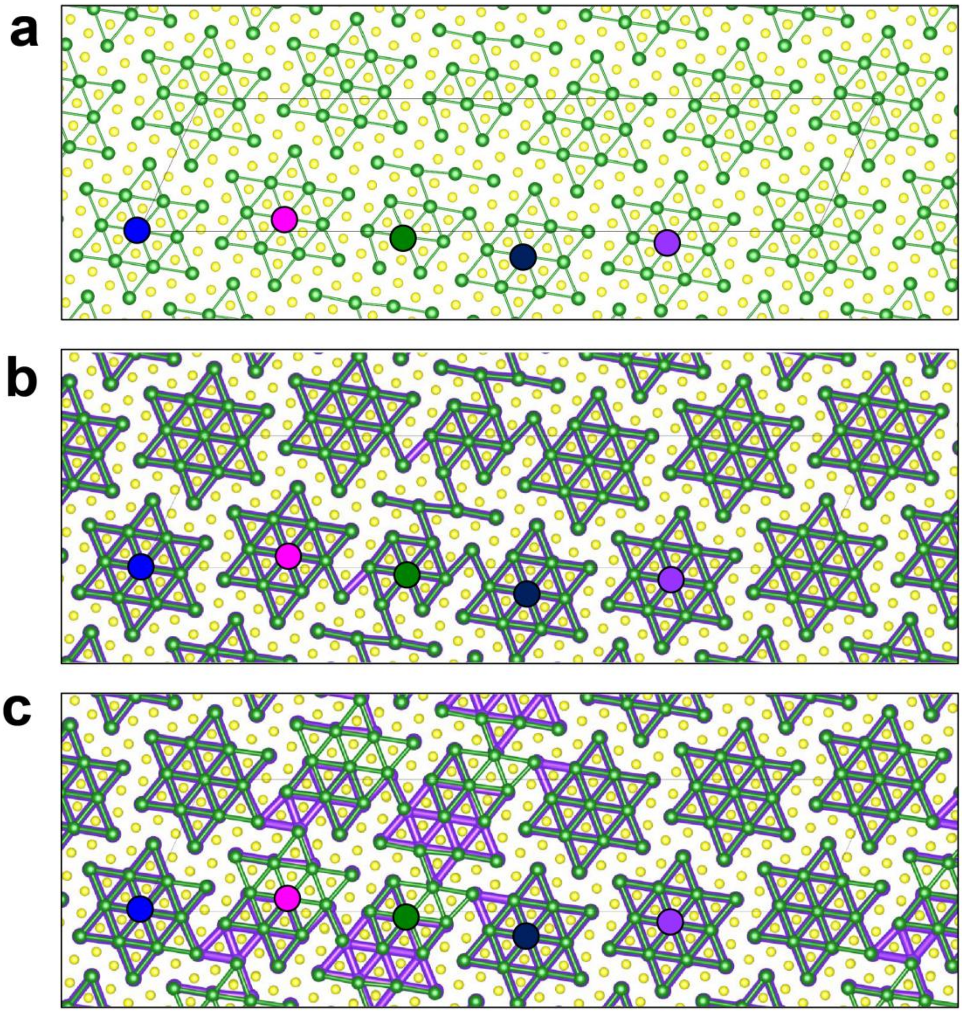

Figure S2. Atomic structure of the DW-2. (a) Single-layer model. (b & c) Bilayer model ((b) without; (c) with a_{CCDW} sliding of sublayer DW). Green and purple balls represent the top and sublayer Ta atoms, respectively. In the single-layer case, the left and right domain DB unitcells have slightly different structural distortions and electronic states since the DW unitcells have asymmetric structure. The present type of DW has 11 electrons per unitcell. While ten of these electrons are paired, one electron at the corner of the unitcell interacts with the right DB unitcell neighboring as shown (a). This makes different distortions of left and right DB unitcells as also shown in the figure. This difference itself is maintained in the bilayer DW model but tends to decrease due to the interlayer coupling. In the bilayer structure without sliding, there is an identical structure of the DW located right below the topmost layer DW. This structure in total has an even number of electrons at the domain wall (11+11) due to the doubled unitcell, eliminating the actively interacting electrons with one DB. Therefore, the bilayer DW induces relatively small distortions in its DB unitcells. In the case of the sliding bilayer structure, the imbalance in symmetry between the first and second layer disrupts the

doubling of unitcells and no longer ensures an even number of electrons, exhibiting properties similar to the single layer case, that is a stronger structural and electronic asymmetry between left and right DB clusters.

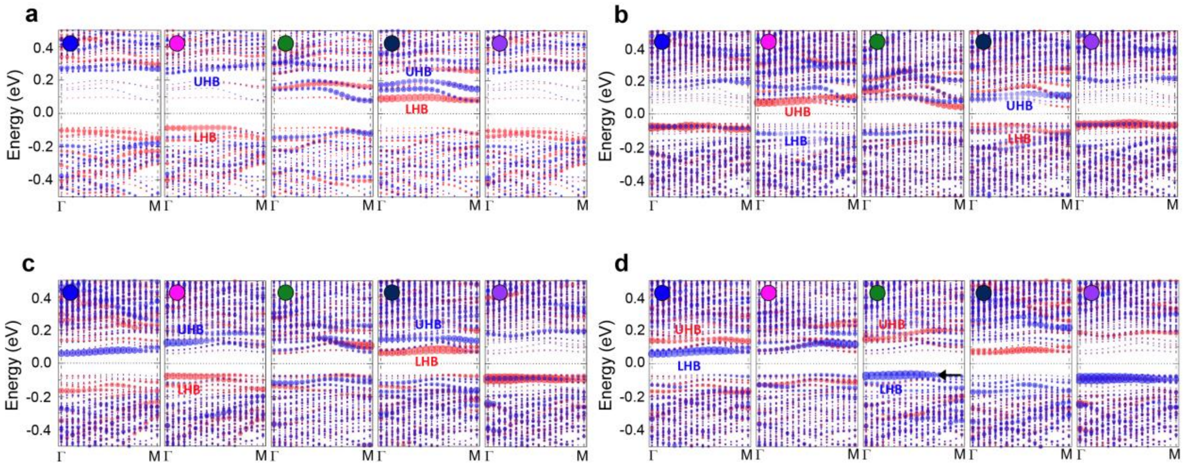

Figure S3. Band structures of the DW-2. (a) Single-layer model. (b-d) Bilayer model. ((b) without; (c & d) with a_{CCDW} sliding of sublayer DW). (b & c) top layer, (d) sub layer. Red and blue circles denote the major and minority spins, respectively. Circle size is proportional to the states localized at corresponding CDW cluster in the Supplementary Figure S2. Arrow in (d) denotes the LHB state of the DS cluster at the sublayer below the DW. The energy level of the LHB corresponds to the energy of the additional in-gap state shown in Fig. 3b, indicating hybridized states with the sublayer.

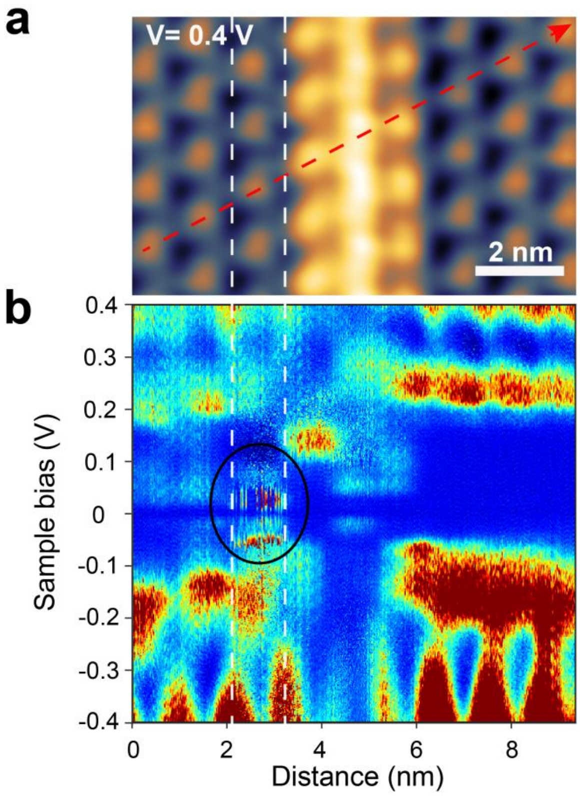

Figure S4. (a) STM topography image of the DW. (b) Line-STS map captured along the red dashed arrow in (a), the in-gap state is marked by the dark circle.

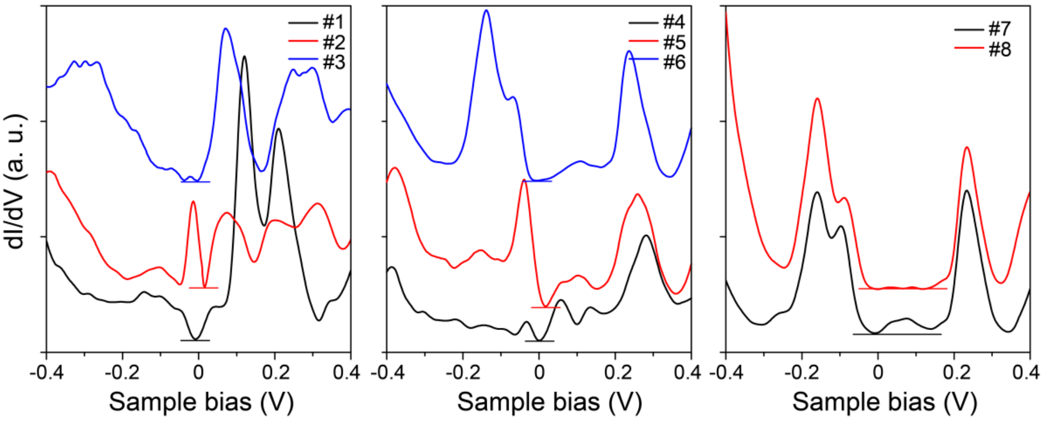

Figure S5. A series of d*I*/d*V* curves taken from the spots #1-8, which are marked in Figure 4a.

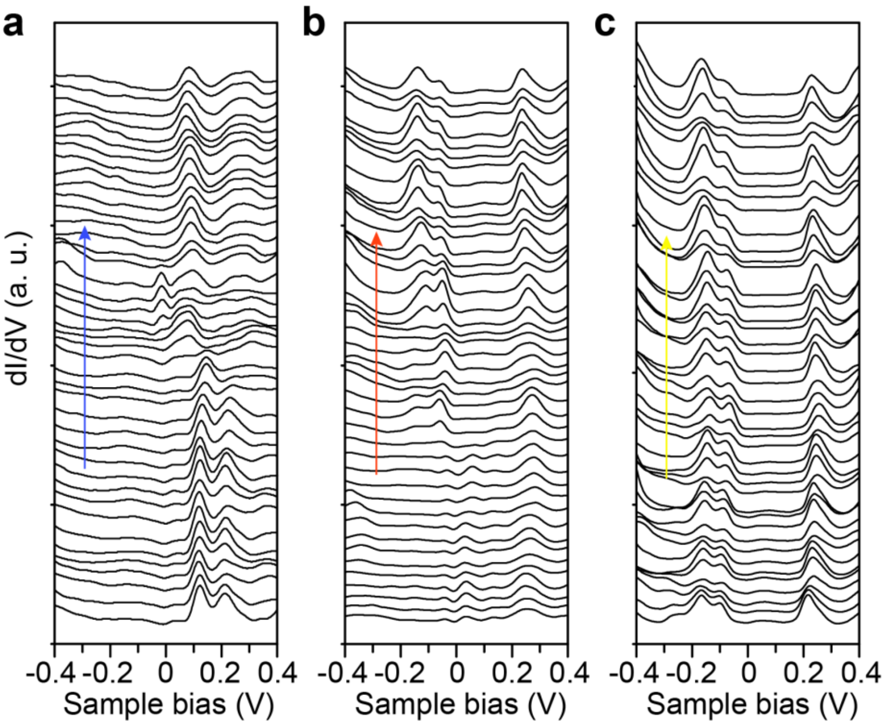

Figure S6. STS spectra along the three arrows in Figure 4a**.**
